# De novo species identification using 16S rRNA gene nanopore sequencing

**DOI:** 10.7717/peerj.10029

**Published:** 2020-10-21

**Authors:** Inga Leena Angell, Morten Nilsen, Karin C. Lødrup Carlsen, Kai-Håkon Carlsen, Gunilla Hedlin, Christine M. Jonassen, Benjamin Marsland, Björn Nordlund, Eva Maria Rehbinder, Carina Saunders, Håvard Ove Skjerven, Anne Cathrine Staff, Cilla Söderhäll, Riyas Vettukattil, Knut Rudi

**Affiliations:** 1 Faculty of Chemistry, Biotechnology and Food Science, Norwegian University of Life Sciences, Ås, Norway; 2 Division of Paediatric and Adolescent Medicine, Oslo University Hospital, Oslo, Norway; 3 Faculty of Medicine, Institute of Clinical Medicine, University of Oslo, Oslo, Norway; 4 Astrid Lindgren Children’s Hospital, Karolinska University Hospital, Stockholm, Sweden; 5 Department of Women’s and Children’s Health, Karolinska Institutet, Stockholm, Sweden; 6 Genetic Unit, Centre for Laboratory Medicine, Østfold Hospital Trust, Kalnes, Norway; 7 Department of Immunology and Pathology, Central Clinical School, Monash University, Melbourne, Victoria, Australia; 8 Department of Dermatology, Oslo University Hospital, Oslo, Norway; 9 Division of Obstetrics and Gynaecology, Oslo University Hospital, Oslo, Norway

**Keywords:** Nanopore, 16S rrNA, Infant gut, Microbiota

## Abstract

Nanopore sequencing is rapidly becoming more popular for use in various microbiota-based applications. Major limitations of current approaches are that they do not enable de novo species identification and that they cannot be used to verify species assignments. This severely limits applicability of the nanopore sequencing technology in taxonomic applications. Here, we demonstrate the possibility of de novo species identification and verification using hexamer frequencies in combination with k-means clustering for nanopore sequencing data. The approach was tested on the human infant gut microbiota of 3-month-old infants. Using the hexamer k-means approach we identified two new low abundant species associated with vaginal delivery. In addition, we confirmed both the vaginal delivery association for two previously identified species and the overall high levels of bifidobacteria. Taxonomic assignments were further verified by mock community analyses. Therefore, we believe our de novo species identification approach will have widespread application in analyzing microbial communities in the future.

## Introduction

Third generation nanopore sequencing has revolutionized the field of analyzing microbial communities, with the promise of on-site high throughput analyses (Acharya et al., 2019). However, despite several recent advances in nanopore sequencing, the error rates are too high for de novo species identification (Shin et al., 2016). Therefore, all current approaches are based on some kind of reference, or black-box systems for species identification (Winand et al., 2019). This severely limits the widespread application of nanopore sequencing in explorative-based applications. In order to exploit the full potential of nanopore sequencing, there is a clear need for de novo approaches for sequence identification.

**Figure 1:**
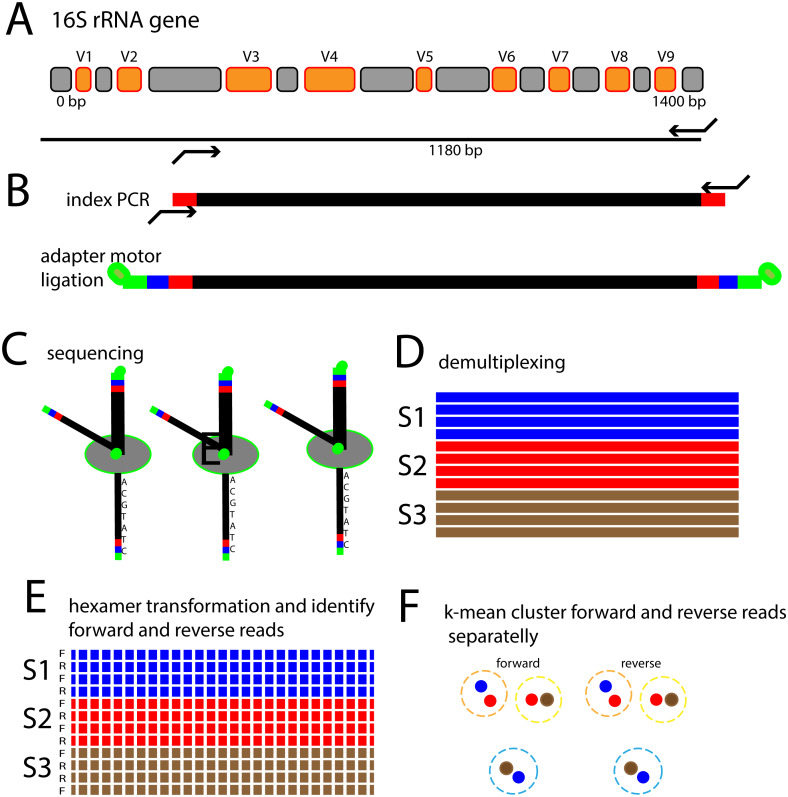
Schematic outline of the 16S rRNA gene nanopore sequencing approach. (A) The V3 to V9 region of the 16S rRNA gene is amplified with PCR primers targeting conserved flanking regions. (B) Indexes for recognizing which sample the sequence is coming from is added in a subsequent PCR step, followed by ligation of adapters containing motor proteins for nanopore sequencing. (C) Sequencing of single DNA molecules through nanopore passage. (D) Identification of which sample the sequences belong to. (E) Identify forward and reverse reads, and transform the sequences to hexamer frequencies. (F) Perform k-mean clustering in order to identify sequence types.

The aim of the present work was therefore to develop an analytical strategy enabling *de novo* identification and quantification of bacterial species using nanopore sequencing. This was achieved through a novel hexamer frequency-based approach in combination with k-means clustering to identify k-mer clustered sequence variants (KSVs). The approach is outlined in Fig. 1.

We evaluated the nanopore de novo species identification approach, both by analyzing the human infant gut microbiota of 3-month-old children and a mock community. The rationale for choosing the infant gut microbiota is that the commonly used black-box nanopore sequencing analytical approach provided by EPI2ME (Oxford Nanopore, Oxford, UK) does not detect *Bifidobacterium* using the standard workflow (Kai et al., 2019). Since *Bifidobacterium* is an important genus in the human infant gut (Avershina et al., 2013), the black-box EPI2ME system cannot reliably be used for human gut microbiota applications.

## Materials and Methods

### Consent by the participants

The PreventADALL study has been approved by the Regional Ethical Committee (REK) for Medical and Health Research Ethics in South-Eastern Norway (2014/518) as well as in Sweden (2015/4:3) by the Regional Ethical Trial Committee of Stockholm. The study is registered as NCT02449850 at clinicaltrial.gov. We received a written consent from all the participants.

### 16S rRNA gene Nanopore sequencing

Genomic DNA from a mock community (HM-783D, BEI Resources, Manassas, Virginia, USA), containing genomic DNA from 20 bacterial strains mixed based on 16S rRNA gene copy number counts, was used as a control and followed the library preparation along with the genomic DNA isolated from the infant fecal samples as described below.

Genomic DNA from 96 fecal samples from 3-month-old children was extracted using the DNA MagMidi kit on the KingFisher Flex robot (Thermo Scientific, USA), following the manufacturer’s recommendations (LCG genomics, UK). 16S rRNA gene amplicons were amplified using a modified CoverAll primer pair (Genetic Analysis, Oslo, Norway) (Casen et al., 2015) tailed with the Oxford Nanopore Technologies (ONT) universal sequences. Each reaction contained 1x HOT FIREPol^®^ Blend Master Mix RTL with 12.mM MgCl2 (Solis BioDyne, Estonia), 0.2 uM of each primer, and 0.1-10 ng DNA template. The following cycling conditions were used: 95 °C for 15 min, followed by 30 cycles of 95 °C for 30 s, 55 °C for 30 s and 72 °C for 1 min 20 s. A clean-up of the amplicons was performed using a 1x volume of AmpureXP beads (Beckman Coulter, USA) following the manufacturer’s recommendations. Next barcoding PCR was performed, using the PCR Barcoding Expansion Pack 1-96 (ONT, UK) where each reaction contained 1x HOT FIREPol^®^ Blend Master Mix RTL with 12.mM MgCl2, 0.2 uM barcode, and 0.5 nM DNA template. Amplification was done at 95 °C for 15 min, followed by 12 cycles of 95 °C for 30 s, 62 °C for 15 s, 65 °C for 2 min before a final elongation step at 65 °C for 10 min. The resulting amplicons were quantified using a Qubit fluorometer (Thermo Fisher Scientific, USA) and pooled together to one library using equimolar concentrations of each sample. The final library was cleaned as described above. DNA repair, end-prep, adapter ligation and clean-up was done using the Ligation Sequencing Kit (SQK-LSK109, ONT, UK) according to the manufacturer’s recommendations.

The sequencing was performed using a R9.4.1 Flow cell (FLO-MIN106) on a MinION sequencing device (ONT, UK) for 24 h. Controlling of the MinION sequencing device and base calling was done using MinKNOW software (ONT, UK). Demultiplexing of barcodes was done using the EPI2ME software (ONT, UK).

The sequencing data has been uploaded in the NCBI SRA database under the BioProject PRJNA637202.

### Sequence processing and analyses

The sequences per sample were first rarefied to 10 000 sequences. In the next step, each sequence was transformed to hexamer frequencies, omitting homopolymer tracts. The hexamer frequency table formed the basis for the identification of KSVs.

We identified *KSVs* using *k-*means clustering. The *k-*means algorithm uses a heuristic approach to find centroid seeds for *k*-means clustering. Details about the clustering algorithm are given by Arthur and Vassilvitskii (Arthur & Vassilvitskii, 2007). We first split the data in two by *k-*means clustering, assuming two clusters, one for the forward reads and one for the reverse reads. For each of the two clusters, we identified KSVs by stepwise increasing the number of clusters until there was no decrease in residual variance. This number was assumed to be the total number of detectable KSVs in the dataset. The taxonomic assignment for each of the clusters was subsequently determined by identifying the closest match in the RDPII database (Cole et al., 2005) for full-length 16S rRNA gene sequences >1,200 bp using the Jaccard similarity index (comparing the presence and absence of hexamers). The assignments were made towards the closest matches in the RDPII database, both for the infant and for the mock data.

For the pairwise  Smith-Waterman sequence alignments (Smith & Waterman, 1981), we used a local reference database consisting only of bacteria included in the mock community, with a taxonomic assignment to the sequences showing the overall highest score in the database. A local database was used due to the extensive computational requirements of alignment-based approaches.

### Statistical analyses

The nonparametric Kruskal–Wallis test was used to compare differences in species abundance between vaginally and c-section delivered children. Pearson correlation was used to determine the association between forward and reverse reads. False discovery was corrected using the Benjamini–Hochberg approach. The statistical analyses were conducted using Matlab version R2019a (MathWorks Inc, Natick, MA, USA) and Minitab version 18 (Minitab Inc, State College, PA, USA).

## Results

### Microbiota composition

We obtained a total of 3,168,160 sequencing reads for 96 samples with an average length of 1,137 bp, totaling 4.6 billion bp of sequence information, with a mean number of reads per sample of 33,002 ± 8,603 [mean ± SD]. The sequences were rarefied to 10,000 reads per sample prior to further analyses. Six samples did not satisfy the rarefying criteria, leaving 90 samples for further analyses.

**Figure 2:**
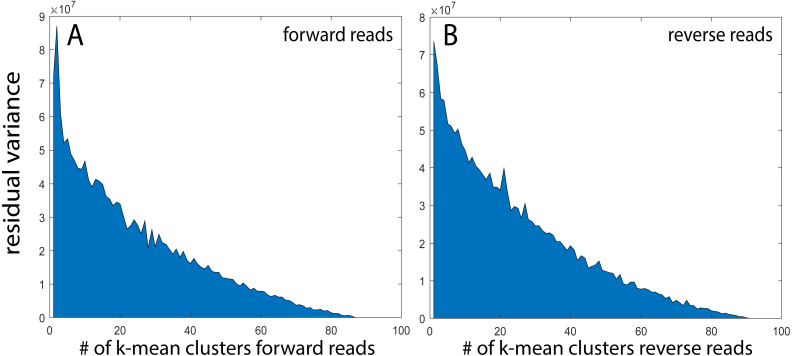
k-mean clustering and taxonomic assignment. (A and B) Residual variance after stepwise k-mean analyses from 2 to 100 clusters.

**Figure 3:**
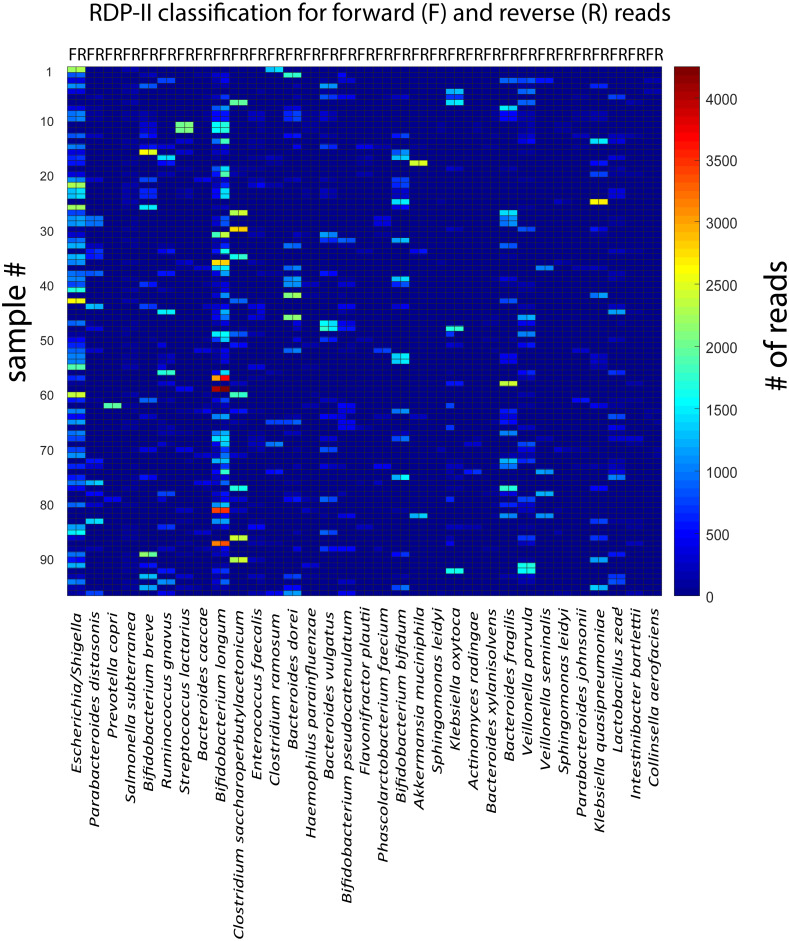
Correlation between forward and reverse and reverse reads for the taxonomically assigned species. The heatmap show the number of forward and reverse reads for all samples (*n* = 96) and identified species (*n* = 33).

For the rarefied samples, the main variation was covered by approximately 92 k-mean KSVs both for the forward and the reverse reads (Figs. 2A and 2B). The matches towards the reference database for taxonomic assignments were also distinct for most of the KSV components (Suppl. Fig. 1). Based on the taxonomic assignments, we identified 33 species with a Pearson correlation >0.9 between forward and reverse reads, showing particularly high abundance of *Bifidobacterium longum* and *Escherichia*. (Fig. 3). The reads belonging to these species accounted for 78.9 ± 13.0% [mean ± std] of all the forward reads, and 84.5 ± 12.5 [mean ± std] of the reverse reads.

### Association with delivery mode

**Figure 4:**
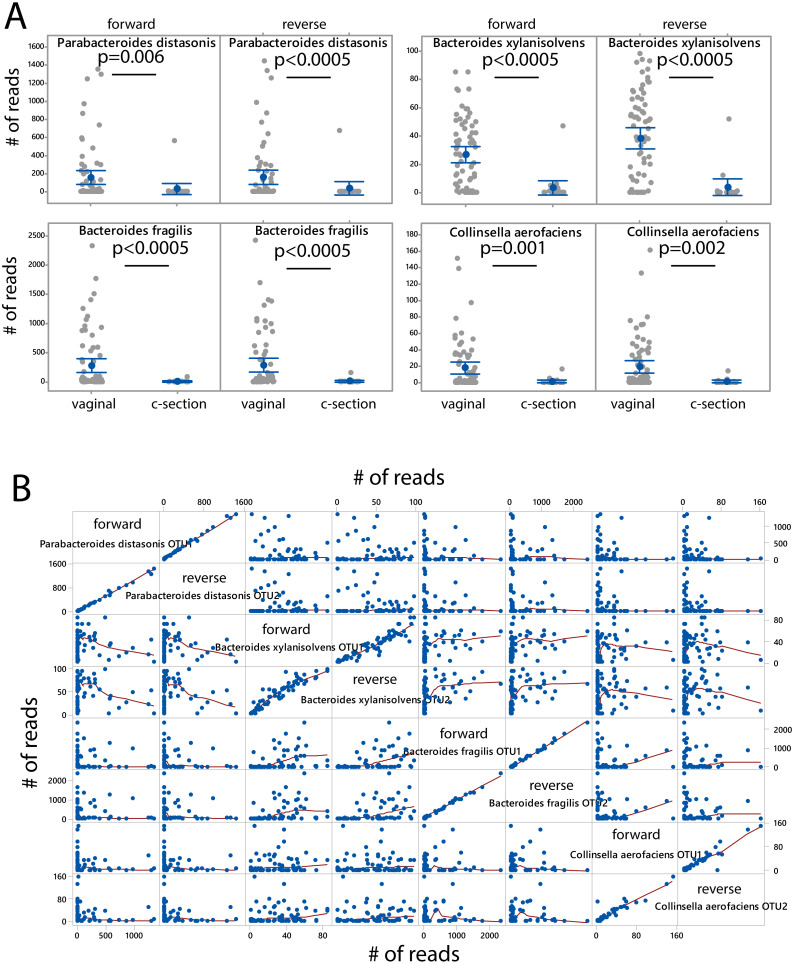
Species associated with vaginal delivery. (A) Comparison of number of sequencing reads for infants delivered vaginally and by c-section. *P*-values were determined the Kruskal–Wallis test. (B) Scatter plot for forward and reverse reads for vaginal delivery associated bacteria.

The microbiota was associated with delivery mode independently for the forward and the reverse reads. After FDR correction (*p* < 0.05) we identified four species showing overrepresentation for vaginal delivery for both forward and reverse reads (Fig. 4A). The number of sequences for these species was also highly correlated and independent of the other species when comparing the forward and reverse reads (Fig. 4B).

### Verification by mock community analyses

**Figure 5:**
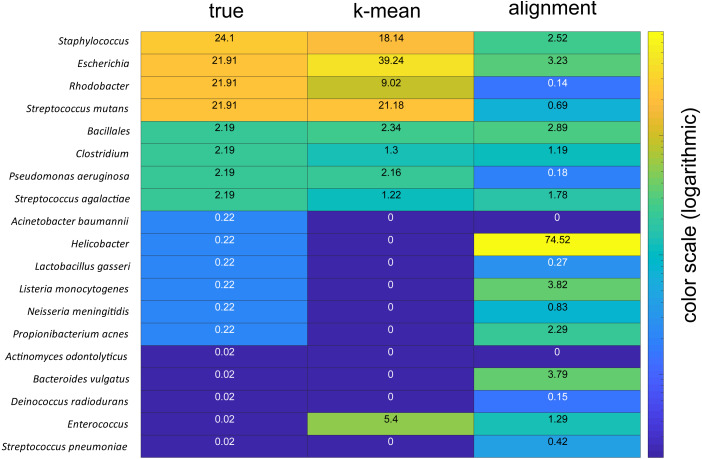
Comparison of mock community analyses. The numbers for the column labeled true represent the given percentages, the column labelled k-mean represent the percentages derived using the k-mean approach, wile column labelled alignment represent the percentages derived based on sequence alignments. The color code is in the logarithmic scale.

The taxonomic assignments were verified by analyses of a mock community with known composition. These analyses showed that the k-mean approach could accurately identify the dominant species in the mock community, while the alignment-based approach failed, severely overrepresenting the AT-rich *Helicobacter* (Fig. 5)*.*

## Discussion

Our study confirms the vaginal delivery association for *Parabacteroides distasonis* and *Bacteroides fragilis* (Shao et al., 2019; Stewart et al., 2018), while *Bacteroides xylanisolvens* and *Collinsella aerofaciens* have not yet been linked to vaginal delivery in the literature. *B. xylanisolvens* is a xylan- degrading bacterium in adults (Chassard et al., 2008) with the potential to induce an IgM response towards glucan antigens (Ulsemer et al., 2016), while *C. aerofaciens* is a proinflammatory gut bacterium that has previously been associated with nonalcoholic fatty liver in adults (Astbury et al., 2020). Since both bacteria are potential immunomodulators in the adult gut, they may play a role in the proper maturation of the immune system of infants (Rodriguez et al., 2015). However, further studies are needed to unveil their potential immunological importance.

The current reference-based 16S rRNA gene nanopore sequence analyses are locked to proprietary databases and primers. This may lead to unforeseen biases such as the lack of bifidobacterial detection from mock samples containing several bacterial species (Kai et al., 2019), and a high error rate with respect to species identification (Winand et al., 2019). Reference-based approaches would therefore both fail to identify the *Bacteroides* species that we identified as being associated with vaginal delivery, and the high levels of *Bifidobacterium* for the 3-month-old children. Furthermore, reference-based approaches lack quality control of the taxonomic assignments. For the k-means KSV approach presented here, the taxonomic assignment for both forward and reverse reads act as quality control for the accuracy of the assignments. Similar distribution based systems have also been used in DADA2 in identifying unique sequence variants from Illumina amplicon sequencing data (Callahan et al., 2016).

Comparison of the taxonomic asignments for the forward and reverse reads revealed that about 80% of the reads showed concordance. Mock community analyses indicated taxonomic identifcation of species present at 20 and 2%, while failing to detect species at the 0.2% and below. There was also an overrepresentation of *Enterococcus*, which we cannot explain*.* Thus, a limitation of the KSV approach could be the identification of species whose abundance is low in all samples. The ∼20% unassigned reads from the main study could therefore partly represent low abundant species.

Poor quality sequences represent a particular challenge with nanopore sequencing data, as opposed to that of the more labor intensive PacBio sequencing (Callahan et al., 2019). However, since the error structure of nanopore sequencing data is blockwise (Magi et al., 2018), we believe using k-mers (Rudi, Zimonja & Naes, 2006) rather than alignment in the taxonomic assignment would be preferable in obtaining high resolution and accuracy. This is also supported by the fact that traditional alignment-based approaches failed in the analyses of nanopore sequencing data.

## Conclusion

By using the KSV approach we provide evidence for de novo species identification and relative quantification using nanopore sequencing. However, further validations by mock community analyses and validation tools, such as TAXCREDIT, are needed before widespread application.

##  Supplemental Information

10.7717/peerj.10029/supp-1 Supplemental Information 1 Similarity to reference sequences in the RDP database The similarities represent Jaccard similarities to the RDP reference database for each of the k-mean clusters both for the forward (A) and the reverse reads (B). Each subplot represent the similarity for a given KSV to each of the full-length sequences in the RDP-II database.

10.7717/peerj.10029/supp-2 Supplemental Information 2 Barcode link sequences to metadata Contains raw reads.

10.7717/peerj.10029/supp-3 Supplemental Information 3 Raw sequence data are linked to metadata through the barcode information

10.7717/peerj.10029/supp-4 Supplemental Information 4 Link between the metadata information with barcode information

## Additional Information and Declarations

### Data Availability

The following information was supplied regarding data availability: The sequencing data is available in the NCBI SRA database: PRJNA637202.
