# Supplementary material for: De novo species identification using 16S rRNA gene nanopore sequencing
Source: PeerJ. 2020 Oct 21;8:e10029. doi: 10.7717/peerj.10029 (PMC7585375; doi:10.7717/peerj.10029)
Supplement: Supplemental Information 1 — The similarities represent Jaccard similarities to the RDP reference database for each of the k-mean clusters both for the forward (A) and the reverse reads (B). Each subplot represent the similarity for a given KSV to each of the full-length sequences in the RDP-II database. [file peerj-08-10029-s001.pdf]

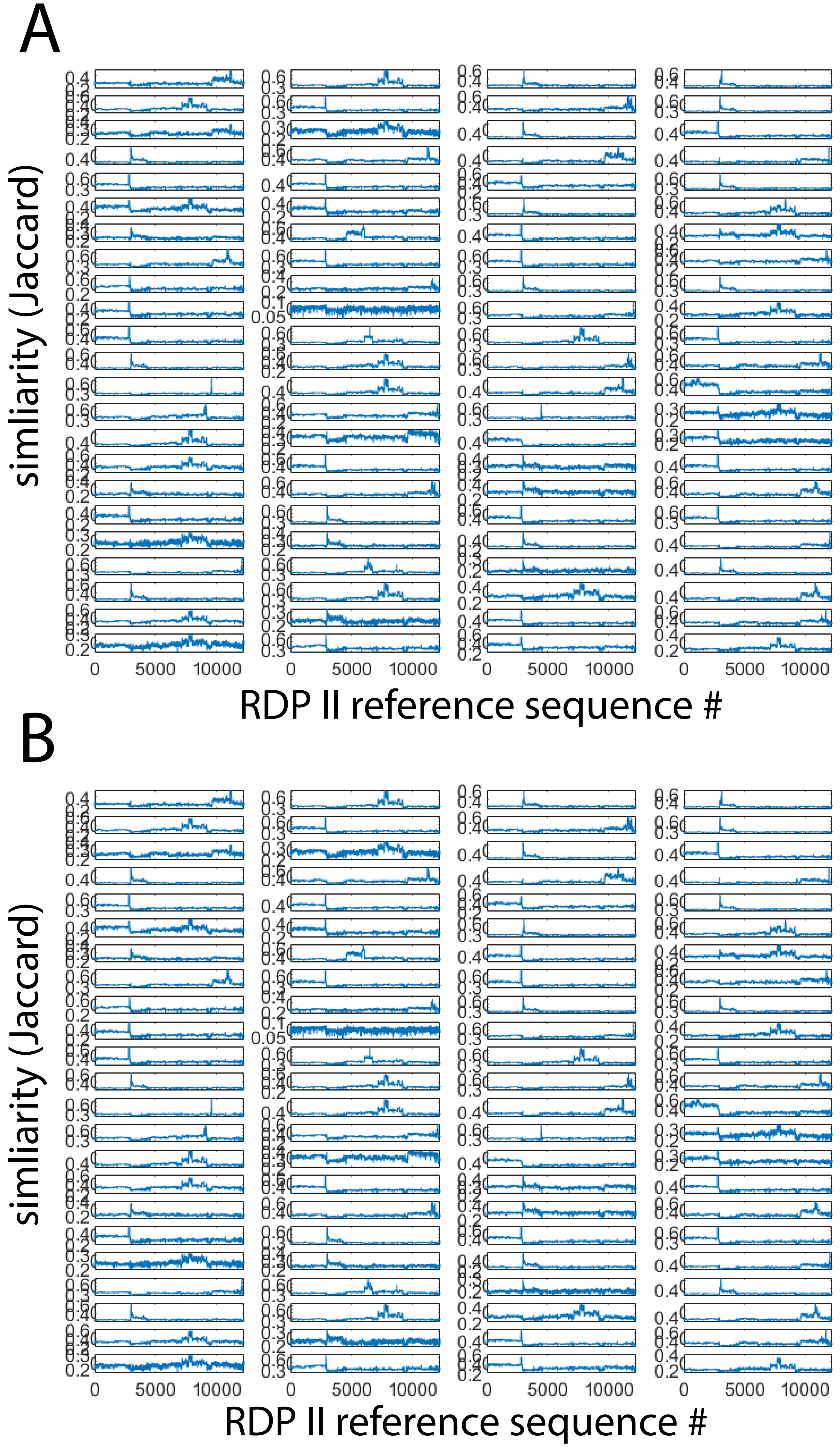
A
simliarity (Jaccard)
0
5000
10000
RDP II reference sequence #
B
simliarity (Jaccard)
0
5000
10000
RDP II reference sequence #
